# Supplementary material for: Actinobacillus pleuropneumoniae Eradication with Enrofloxacin May Lead to Dissemination and Long-Term Persistence of Quinolone Resistant Escherichia coli in Pig Herds
Source: Antibiotics (Basel). 2020 Dec 15;9(12):910. doi: 10.3390/antibiotics9120910 (PMC7765418; doi:10.3390/antibiotics9120910)
Supplement: Supplementary file 1 [file antibiotics-09-00910-s001.pdf]

antibiotics

MDPI

Supplementary Materials

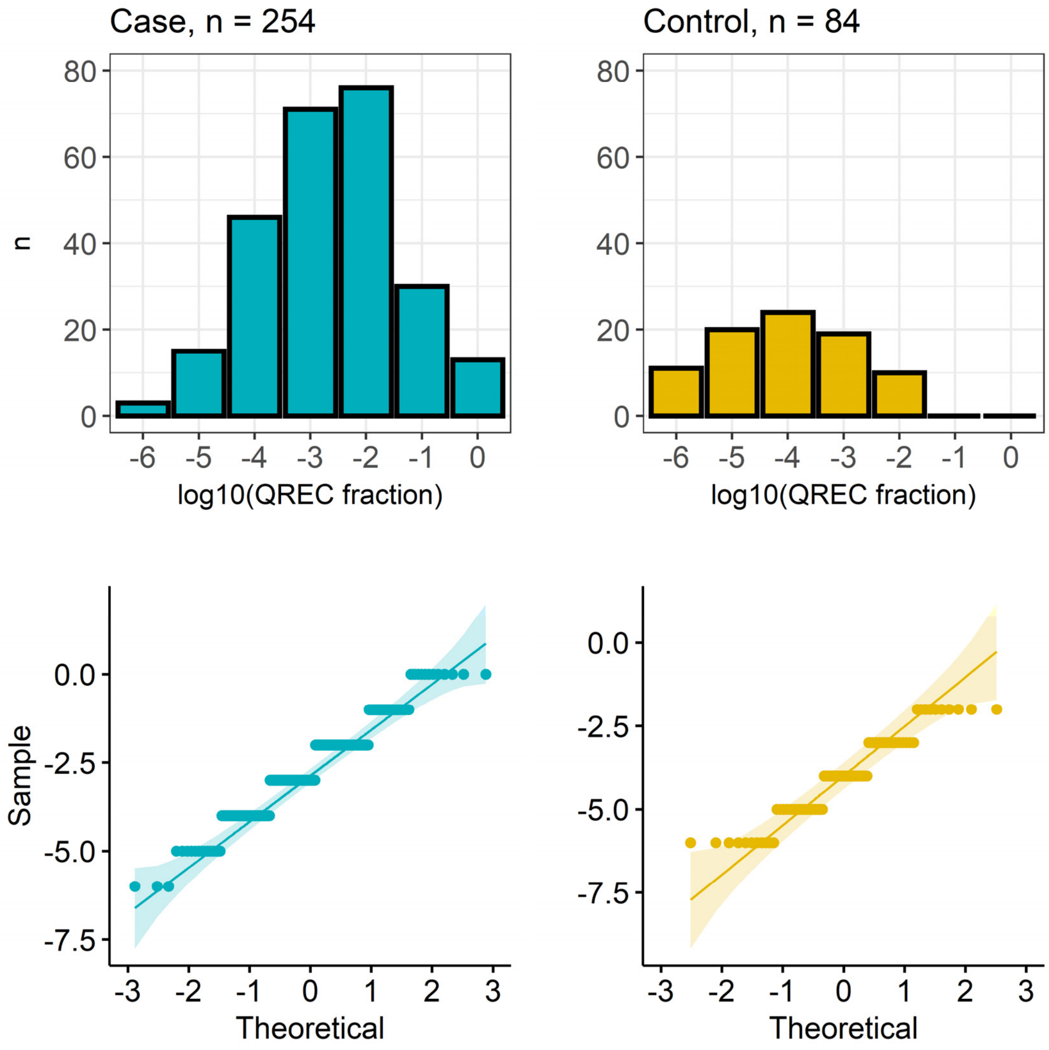

Figure S1. Distribution of the log10-transformed fractions of quinolone resistant *E. coli* (QREC) isolates in the case (blue) and control (yellow) herds, showing a normal distribution.

Table S1. X^2-tests for all four age groups, case versus control.

**Age group**	**Number of samples**			**X^2-value**	***p*-value**
	Type	Positive	Negative		
Weaner	Case	72	28	103.96	$p < 0.01$
	Control	3	100		
Fattening pig	Case	25	35	21.068	$p < 0.01$
	Control	7	73		
Gilt	Case	81	20	49.121	$p < 0.01$
	Control	26	62		
Sow	Case	76	26	13.259	$p < 0.01$
	Control	48	49		

Table S2. Resistance patterns among the 130 quinolone resistant *E. coli* isolates with additional resistances in addition to ciprofloxacin and/or nalidixic acid. AMP = ampicillin, CAZ = ceftazidime, CHL = chloramphenicol, CTX = cefotaxime, GEN = gentamicin, SMX = sulfamethoxazole, TET = tetracycline, TGC = tigecycline, TMP = trimethoprim.

Antimicrobial	Number of isolates	Number of resistances
TET	62	1
SMX, TET	36	2
SMX, TMP, AMP	10	3
SMX, TMP, TET, CHL, AMP, GEN	6	6
GEN	4	1
SMX, AMP	3	2
SMX, TET, CHL, AMP, GEN	2	5
TGC	2	1
AMP	1	1
SMX	1	1
SMX, CAZ, CTX,	1	3
SMX, TET, AMP, GEN	1	4
TET, TGC	1	2

Table S3. Overview of the relative amount of bacteria detected if growth at the respective dilution. The numbers were used for both QREC and total *E. coli*.

Dilution	No. of bacteria
Overnight culture	10^{3}
10^{-1}	10^{4}
10^{-2}	10^{5}
10^{-3}	10^{6}
10^{-4}	10^{7}
10^{-5}	10^{8}
10^{-6}	10^{9}

Publisher's Note: MDPI stays neutral with regard to jurisdictional claims in published maps and institutional affiliations.

© 2020 by the authors. Licensee MDPI, Basel, Switzerland. This article is an open access article distributed under the terms and conditions of the Creative Commons Attribution (CC BY) license (http://creativecommons.org/licenses/by/4.0/).
